# The Detection of Porphyromonas Gingivalis in Geriatrics and Its Associated Periodontal and Clinical Factors

**DOI:** 10.7759/cureus.79522

**Published:** 2025-02-23

**Authors:** Aina Nabilah Suhaimi, Yashdev Atri Roop Kishore, Mohd Shaiful Ehsan Shalihin, Hairul Aini Hamzah, Sulhi Abidin, Edre Mohammad Aidid, Ramli Musa

**Affiliations:** 1 Department of Health Sciences, International Islamic University Malaysia, Kuantan, MYS; 2 Department of Kulliyyah Medicine, International Islamic University Malaysia, Kuantan, MYS; 3 Department of Family Medicine, International Islamic University Malaysia, Kuantan, MYS; 4 Department of Basic Medical Science, International Islamic University Malaysia, Kuantan, MYS; 5 Department of Prosthodontics, International Islamic University Malaysia, Kuantan, MYS; 6 Department of Community Medicine, International Islamic University Malaysia, Kuantan, MYS; 7 Department of Psychiatry, International Islamic University Malaysia, Kuantan, MYS

**Keywords:** cognitive impairment, diabetes, geriatrics, periodontitis, porphyromonas gingivalis

## Abstract

Background: *Porphyromonas gingivalis* is one of the important pathogens in the initiation and progression of periodontitis. The findings regarding the bacterium and periodontal status in geriatric subjects are not widely reported. Bacterial analysis is needed to provide more insight regarding the severity of the disease. This study evaluated the presence of *P. gingivalis* in the oral cavity of geriatric patients and found a significant association between *P. gingivalis* infection and medical illness.

Methods: Periodontal pocket samples were obtained from 32 geriatrics who were chosen randomly from health clinics. Clinical periodontal parameters were recorded during the oral examination. Samples were subjected to DNA extraction and polymerase chain reaction (PCR) amplification. The identification of *P. gingivalis* by PCR assay was determined based on the *P. gingivalis *16S rRNA subunit amplification. Medical illnesses of patients were recorded. Mild cognitive impairment was diagnosed according to cognitive score assessment and daily function.

Results: Approximately 90.63% (n=29) of geriatric patients had periodontitis. The presence of *P. gingivalis* was significantly associated with periodontal depth and clinical attachment loss (p = 0.037). The extension of periodontal disease, cognitive impairment status, and diabetes status are highly associated with *P. gingivalis.*

Conclusion: Due to asymptomatic periodontal disease in the early phase, early screening of bacterial detection and oral public health awareness is crucial, especially among elderly groups who are smokers and diabetics. *P. gingivalis* infection is associated with worsening geriatric periodontitis. Adequate oral care and comorbidity control among geriatrics are indeed crucial to preventing disease progression. It is important to improve and modify the local diabetic checklist protocol for geriatric diabetic patients by incorporating oral health and oral microbiome assessments, as well as cognitive assessments.

## Introduction

Advancements in modern medicine, medical technologies, and pharmacological discoveries have significantly increased human life expectancy, enabling more individuals to live beyond 60 years. According to the World Health Organization (WHO), the global population aged 60 years and older was approximately 1 billion in 2020 and is projected to reach 1.4 billion by 2030, doubling to nearly 2.1 billion by 2050. By the year 2100, older adults are expected to constitute nearly one-quarter of the global population, raising concerns about the rising burden of infections, non-communicable diseases (NCDs), and mortality [1,2].

Aging is accompanied by immunosenescence, a gradual decline in immune function, which increases susceptibility to various infections, including oral infections [3]. Epidemiological data indicate that nearly all geriatric individuals have experienced dental caries, and two-thirds suffer from gingivitis or periodontitis. Alarmingly, 23% of these cases progress to complications such as neurocognitive decline and other systemic conditions, including diabetes and hypertension [4,5]. Periodontitis, primarily caused by *Porphyromonas gingivalis*, has been strongly linked to severe systemic infections such as sepsis, pericarditis, encephalitis, and cognitive impairment. Without adequate treatment, periodontitis significantly increases morbidity and mortality rates, thereby exacerbating the burden on caregivers, families, and healthcare systems [6].

Periodontal disease in the geriatric population

Periodontitis is among the most prevalent oral infections, with epidemiological studies reporting an 82% prevalence among elderly populations [7]. Given the increasing number of older adults worldwide, the prevalence of periodontitis is expected to rise further in the coming years. However, despite its global significance, periodontal disease data in the WHO Oral Health Data Bank remains scarce, with only 18 countries reporting statistics on geriatric periodontal health [7]. While several epidemiological studies have surveyed periodontitis, most are limited to specific countries or local settings [8]. For instance, the Global Burden of Disease Study 2021 highlights the prevalence and severity of periodontitis but lacks insights into its bacterial etiology [8].

There is an urgent need for comprehensive research on periodontitis in the geriatric population, particularly regarding its microbial causative factors. Increasing awareness among both the public and primary healthcare providers is crucial to recognizing *P. gingivalis* as a key pathogen in periodontitis. Emphasizing oral health education during routine geriatric healthcare visits can enhance early detection and management, mitigating the risk of systemic complications. This study aims to bridge this knowledge gap by investigating *P. gingivalis* in the local geriatric population and underscoring its clinical relevance in routine medical practice [9,10].

Role of* P. gingivalis* in periodontitis and systemic diseases

Approximately 10 to 30 bacterial species contribute to the progression of periodontitis, with the most dominant being the Gram-negative anaerobic bacteria collectively referred to as the "red complex bacteria" (*P. gingivalis, Tannerella forsythia, *and *Treponema denticola*). Among these, *P. gingivalis* is the most prevalent species, detected in 86% of periodontal pockets, and is implicated in disease progression from initial to advanced periodontitis [9].

Beyond its role in periodontitis, *P. gingivalis* has been extensively linked to systemic diseases, including Alzheimer’s disease, which can further impair daily functioning and quality of life in elderly individuals [6,10]. Emerging evidence has demonstrated strong associations between *P. gingivalis* and comorbidities such as ischemic heart disease, oropharyngeal and gastrointestinal malignancies, nonalcoholic fatty liver disease, and rheumatologic disorders. These associations arise from the ability of *P. gingivalis* to colonize distant organs, triggering chronic inflammation and immune dysregulation [6].

Detection methods and study objectives

Accurate and early detection of *P. gingivalis* is essential for effective disease management. Traditional culture-based methods, although considered the gold standard, are time-consuming, requiring up to 14 days for bacterial identification and demonstrating low sensitivity. To overcome these limitations, polymerase chain reaction (PCR) assays have been developed as a more rapid and sensitive diagnostic tool [11]. This study aims to 1) identify *P. gingivalis* using a PCR-based detection protocol targeting the 16S rRNA subunit, 2) evaluate the association between *P. gingivalis* presence and clinical periodontal parameters in geriatric patients, and 3) assess the correlation between* P. gingivalis* and systemic comorbidities commonly observed in the elderly. By addressing these objectives, this study seeks to enhance clinical understanding of periodontitis in geriatrics, promote early detection strategies, and reinforce the need for integrated oral-systemic healthcare approaches. Appendix (Figure 2) shows the PCR performance protocol.

## Materials and methods

Subjects and clinical periodontal screening

This analytical cross-sectional study was conducted at the start of 2022. All the human subject protocols were approved by the Medical Research and Ethics Committee (NMRR ID-21-02107-4AY (IIR)) and IIUM Research Ethics Committee (IREC 2021-266). Written informed consent from patients was obtained before the oral examination and sample collections commenced.

This study involved 32 geriatric patients recruited from government health clinics. Two clinics were randomly selected from a pool of eight using Microsoft Excel’s (Redmond, USA) randomization function, with selection criteria prioritizing those with the highest geriatric attendance in the district. Patient recruitment was conducted via systematic random sampling from existing clinic records, whereby every third geriatric patient was approached until the minimum sample size was achieved. The final sample comprised approximately 15 to 20 patients per clinic, with proportional allocation based on the annual geriatric attendance ratio at each facility relative to the total district-wide attendance. The minimum sample size was determined based on previous studies on sample size requirements for the association, whereby at least 30 samples are required (power 80) to measure the association, and at least 20 respondents are required for the Fisher’s exact or chi-square tests [12].

The patients were ≥ 60 years old. Individuals who were edentulous or without periodontal pockets were excluded, as these factors are strongly linked to severe periodontitis and could introduce data bias. Patients who had taken antibiotics in the past three months, as well as those with overt dementia or an inability to respond to questionnaires, were also excluded. The patients’ clinical and sociodemographic data, including age, gender, smoking status, and the presence of non-communicable diseases, were recorded, as determined by blood pressure and blood sugar records. Mild cognitive impairment (MCI) was assessed using the validated Malay version of the Elderly Cognitive Assessment Questionnaire. This version is an interview-based measure with high sensitivity and specificity, consisting of three sections: memory, orientation and information, and memory recall. The questionnaire has a maximum score of 10 and a minimum score of 0, with a cut-off point of 7 or lower indicating cognitive impairment. Mild cognitive impairment (MCI) was defined as a score of 7 or below on the Malay version of the ECAQ Questionnaire, along with an intact Barthel Index score and no other causes as assessed clinically by the physician.

The periodontal status of each geriatric patient was examined by a dental specialist, and they were classified into three groups based on periodontal health, periodontal probing depth (PPD), bleeding on probing, and clinical attachment loss [13]. Group I included patients with healthy periodontal status, characterized by PPD ≤ 3 mm, no inflammation, no bleeding after probing, and no clinical attachment loss (n = 3). Group II consisted of patients with initial to moderate periodontitis, having PPD ≥ 4 mm, along with inflammation and bleeding after probing, as well as early tooth mobility. Group III included those with severe periodontitis, with PPD ≥ 5 mm, also exhibiting inflammation and bleeding after probing as well as multiple missing teeth [13].

Subgingival sample collection

Six-point periodontal charting was conducted on all teeth present in the mouth to identify suitable sites for sample collection. A periodontal probe with William’s marking was used to measure the periodontal pocket depth, and the actual depth of each patient was recorded. Sterile paper points were placed into the bottom of the periodontal pocket for a few seconds. Then, the paper points were transported in a sterile Eppendorf tube (Hamburg, Germany) containing 1.5 mL of anaerobic Cary Blair (Mantacc, Guangdong, China) transport medium. In 4ºC of a transfer medium, all samples were sent to the microbiology laboratory for DNA extraction and polymerase chain reaction (PCR) analysis.

DNA extraction

The ATCC 33277 strain was used as a standard control in this study. The strain was grown anaerobically in brain heart infusion (BHI) broth with the addition of 5 mg/L of hemin and 5 mg/L of menadione and placed in an anaerobic incubator for four days at 37ºC. The culture was subjected to DNA extraction and PCR amplification.

The bacterial DNA extraction was done based on the manufacturer’s protocol of the Purelink Microbiome extraction kit (Invitrogen, US). The DNA from each clinical sample was extracted with the same protocol.

PCR amplification 

The PCR reaction was done in a total volume of 25 µl containing 12.5 µl of the PCR master mixture (containing Taq DNA polymerase, dATP, dGTP, dCTP, dTTP, and MgCl2), 1 µl of each forward and reverse primer, 10 µl of nuclease-free water, and 0.5 µl of the extracted DNA. A set of primers, P1 (forward): 5’AGGCAGCTTGCCATACTGCG 3’, and P2 (reverse): 5’ ACTGTTAGCAACTACCGATGT 3’were used. Nuclease-free water was used as a negative control, replacing the extracted DNA. The initial denaturation was at 50ºC for two minutes, followed by 30 cycles for 30 seconds. The annealing temperature was at 58ºC for 30 seconds. The elongation temperature was at 72ºC for one minute, followed by a final extension for 10 minutes. The expected was 404 bp. The primers and PCR conditions employed in this study are summarized in Appendix (Table 6).

Agarose gel electrophoresis

The PCR products were analyzed on 1.5% of the agarose gel. About 5 µl of DNA samples with 2 µl of loading dye were loaded into each well of agarose gel carefully with a standard 50 bp ladder. The power supply was set up at 100 V and switched on for 30 minutes. The gel was analyzed under an ultraviolet transilluminator at 254 nm, and the picture was captured.

Statistical analysis

The results were statistically analyzed by using IBM Corp. Released 2011. IBM SPSS Statistics for Windows, Version 20.0. Armonk, NY: IBM Corp. Fisher’s exact test was applied to find out the significant correlation between the presence of* P. gingivalis* with different periodontal status in three different geriatric groups. The U Mann-Whitney test, bivariate analysis, and multiple logistic regression were applied to determine if there is a statistically significant association between the periodontal parameters and clinical comorbidities with the presence of *P. gingivalis*. The finding was considered statistically significant if the p-value was ≤ 0.05.

## Results

**Figure 1:**
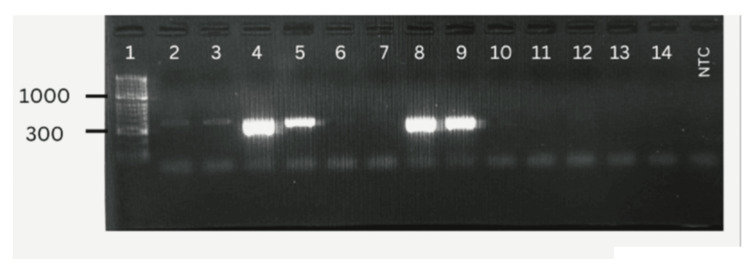
Positive and negative detection of P. gingivalis in clinical samples. Lane 1: DNA ladder (50 bp). Lanes 2, 3, 4, 5, 8, and 9 showed positive detection of *P. gingivalis* (around 404 bp size). Lanes 6, 7, 10, 11, 12, 13, and 14 showed negative detection of *P. gingivalis*. Lane 15 is the negative template control (NTC).

**Figure 2:**
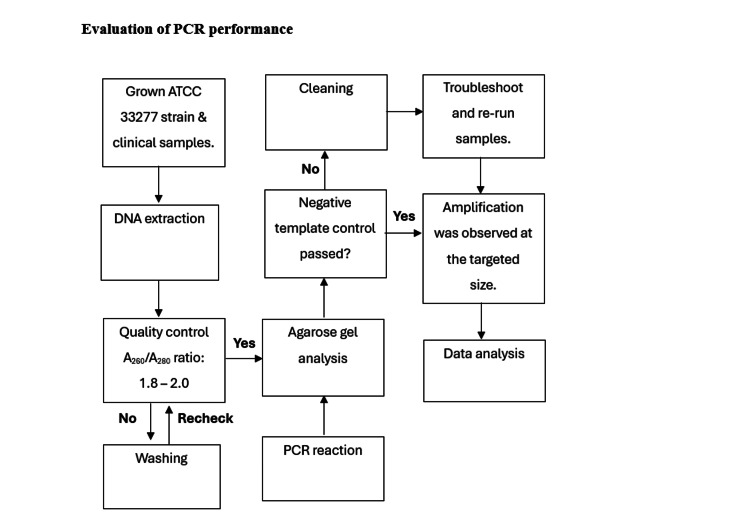
PCR performance protocol

PCR amplification of each clinical sample was done in triplicate to ensure accuracy and avoid false-positive or false-negative outcomes. Figure 1 shows a few positive* P. gingivalis* detections in the clinical samples.

The demographic data of the respondents are presented in Table 1. The majority are male geriatrics who are hypertensive and diabetic. About 90.63% (n=29) of 32 geriatric patients have periodontitis, and only 9.38% have normal periodontal conditions with no inflammation, a PPD ≤ 3 mm, no bleeding on probing, and no clinical attachment loss. We found that the severity of PPD is associated with the presence of *P. gingivalis*. No *P. gingivalis* is observed at the sites with PPD ≤ 3 mm. However, *P. gingivalis* was identified at 100% in severe periodontitis patients with PPD ≥ 5 mm. The significant correlation by Fisher’s exact test is summarized in Table 2.

**Table 1:** Descriptive analysis of demographic variables

Measure	Total number (N)	Percentage (%)
Gender		
Male	20	62.50
Female	12	37.50
Total	32	100.00
Age group		
60 – 70 years old	19	59.37
71 – 80 years old	12	37.50
≥ 81 years old	1	3.13
Total	32	100.00
Ethnicity		
Malay	26	81.25
Chinese	4	12.50
Indian	2	6.25
Total	32	100.00
Smoking status		
Smokers	11	34.36
Non-smokers	21	65.64
Total	32	100.00
Hypertensive		
Yes	32	100.00
No	0	0
Total	32	100.00
Diabetics		
Yes	28	87.50
No	4	12.50
Total	32	100.00

**Table 2:** Detection of P. gingivalis in geriatric subjects according to oral health status PPD: periodontal pocket depth *P-value is significant if < 0.05

Geriatric groups	Prevalence	P. gingivalis present
I – Healthy (PPD ≤ 3 mm)	3 (9.38%)	0 (0%)
II – Chronic Periodontitis (PPD ≥ 4 mm)	25 (78.13%)	15 (46.88%)
III – Chronic periodontitis (PPD ≥ 5 mm)	4 (12.50%)	4 (100%)
Fisher’s exact test		p-value = 0.035*

When comparing the distribution of *P. gingivalis* among genders, male geriatrics (78.95%, n=17) with periodontitis more frequently have *P. gingivalis* infection than female geriatrics at 40%(n=4). From all geriatric patients with chronic periodontitis, a statistically significant association is noted between PPD and periodontal clinical attachment loss (CAL) in the presence of *P. gingivalis*. Meanwhile, no significant association is observed between the plaque score (PS) and bleeding on probing (BOP). The extension of the disease with the presence or absence of* P. gingivalis* is summarized in Table 3.

**Table 3:** Association between clinical periodontal parameters and P. gingivalis detection *Values representing the mean and standard deviation *P-value is significant if < 0.05.

Clinical parameter	P. gingivalis present	P. gingivalis absent	Mann-Whitney U Test p-value
Plaque score (%)	35.37 ± 26.00	19.16 ± 15.35	0.077
Bleeding on probing (%)	34.15 ± 0.28	18.71 ± 16.86	0.157
Probing depth (mm)	4.68 ± 0.94	3.25 ± 1.87	0.037*
Clinical attachment loss (mm)	0.65 ± 0.90	0.25 ± 0.23	0.037*

*P. gingivalis* is also noted to be significantly associated with diabetes, mild cognitive impairment illness, and smoking status, as shown in Tables 4, 5.

**Table 4:** Bivariate analysis of P. gingivalis with demographic and clinical profiles P-value is significant if < 0.05.

Variables	P. gingivalis		Total	P-value Chi-Square*
	Present	Absent		
Gender				
Male	18	3	21	
Female	3	8	11	
Total	21	11	32	0.002
Diabetes				
Yes	20	1	21	
No	5	6	11	
Total	25	7	32	0.003
MCI				
Yes	20	5	25	
No	1	6	7	
Total	13	11	32	0.003
Smoker				
Yes	12	9	21	
No	1	10	11	
Total	13	19	32	0.010

**Table 5:** Multiple logistic regression of factors associated with P. gingivalis infection P-value < 0.05 is significant.

P. Gingivalis		Odd ratio	Std. Error	Wald	df	Sig.	95% Confidence Interval for Exp(B)	
							Lower Bound	Upper Bound
	Intercept	-25.463	1.095	540.323	1	< .001		
	Diabetic	23.854	1.549	237.089	1	< .001	1098990003.017	476809894956.808
	MCI	23.854	.000	.	1	< .001	22891249592.306	22891249592.306

## Discussion

Periodontitis is an irreversible inflammatory disease of the periodontal tissues caused by bacterial accumulation, leading to tissue penetration and eventual tooth loss [13]. This condition significantly impacts the overall health of elderly individuals, as it can cause difficulties in speaking, chewing, and nutrition intake, subsequently reducing their quality of life [14]. Studies have established strong associations between periodontitis and various chronic systemic diseases, including diabetes, Alzheimer’s disease, rheumatoid arthritis, and cardiovascular disease, with *Porphyromonas gingivalis *(*P. gingivalis*) identified as a key etiological agent contributing to disease progression [6,15-17].

Prevalence of *P. gingivalis* and its clinical implications

Previous research has compared the prevalence of periodontitis across different age groups, with evidence suggesting that the chronicity of periodontal disease increases with age [18]. The current study focuses on oral screening among the elderly population to determine the frequency of *P. gingivalis* detection and its association with clinical periodontal parameters. Periodontal disease diagnosis primarily relies on clinical attachment loss (CAL) and probing pocket depth (PPD), both of which are strongly linked to periodontopathogenic bacterial invasion [19]. Combining bacterial analysis with clinical periodontal parameters can provide a more comprehensive understanding of disease progression [18].

The present study revealed a high prevalence of periodontitis among geriatric patients, with 90.63% (n=29) affected, while *P. gingivalis* was detected in 65.52% (n=21). The prevalence of *P. gingivalis* in the periodontitis group closely aligns with findings by Joshi et al. in Indian subjects (66%) [20]. Other studies in Indian populations reported even higher prevalence rates, with Kulkarni et al. observing 76.67% and Kugaji et al. reporting 79.16% [21]. Notably, in contrast to other studies, this study found no detection of *P. gingivalis* in healthy subjects, whereas previous research reported at least a 10.00% prevalence in healthy individuals [21].

Association between* P. gingivalis* and periodontal parameters

A Mann-Whitney U test revealed a statistically significant association between the presence of *P. gingivalis* and both PPD and CAL (p=0.037). These results are consistent with findings by Kumawat et al., who reported a significant association (p<0.01) [22]. However, Joshi et al. found no significant correlation between *P. gingivalis* and PPD [20], and Sazan et al. reported no significant association between *P. gingivalis* and CAL [23]. While the present study did not find significant correlations between *P. gingivalis* and plaque score (PS) or bleeding on probing (BOP), previous studies have reported significant associations between *P. gingivalis* and both parameters [22,24]. These discrepancies could be attributed to differences in sample size, study populations, sampling techniques, detection methods, and demographic factors [18].

Impact of smoking and comorbidities on *P. gingivalis* infection

Analysis of the clinical background of geriatric patients (Table 4) indicated a strong association between smoking and *P. gingivalis* infection (p=0.010). Male geriatric patients exhibited a higher infection rate (78.95%, n=17) compared to females (40%, n=4), likely due to higher smoking prevalence among men. Smoking has been widely recognized as a key factor in the colonization of pathogenic bacteria and the progression of periodontal disease [25].

This study also highlights a significant association between *P. gingivalis *infection and comorbidities such as diabetes, hypertension, and mild cognitive impairment (MCI), with prevalence rates of 87.50%, 100%, and 80%, respectively (Table 4) [25,26]. Given these findings, it is crucial to control risk factors such as hyperglycemia and smoking cessation in geriatric populations to improve oral health and prevent *P. gingivalis* infections from contributing to systemic diseases. Adequate eradication of *P. gingivalis* may also help mitigate the progression of related systemic illnesses and cognitive impairment [6].

*P. gingivalis*, cognitive decline, and neurodegeneration

Multiple logistic regression analysis (Table 5) identified diabetes and MCI as the only significant factors associated with *P. gingivalis* presence. Research suggests that *P. gingivalis*-induced periodontitis exacerbates cognitive impairment in diabetic individuals, reinforcing the link between systemic inflammation and neurodegeneration [27]. The oral microbiome plays a crucial role in cognitive decline, with *P. gingivalis* strongly correlating with MCI [28].

Recent studies on Parkinson’s disease (PD) and MCI have demonstrated alterations in oral microbiota composition, with *P. gingivalis* more frequently detected in cognitively impaired patients, indicating a potential association between oral dysbiosis and neurodegenerative diseases [29]. Furthermore,* P. gingivalis* has been identified as a key factor in PD-related cognitive impairment, underscoring the role of oral infections in neuroinflammation and neurodegeneration [30].

Clinical and public health implications

These findings emphasize the importance of oral health as a modifiable risk factor in preventing cognitive decline, particularly in diabetic individuals. Future research should explore the mechanistic pathways linking *P. gingivalis* to metabolic and neurodegenerative disorders to inform targeted interventions for at-risk populations.

Since *P. gingivalis* primarily colonizes deeper alveolar pockets, effective eradication requires deep oral cleansing and regular mouthwash use, as superficial tooth brushing alone is insufficient. Incorporating oral health and microbiome assessments into local diabetic checklist protocols for geriatric patients could enhance early detection of MCI, ultimately improving the quality of life among elderly populations.

Study limitations and future directions

Despite exceeding the minimum required sample size, the study’s relatively small cohort limits the generalizability of findings. Future studies with larger sample sizes would provide a more robust impact analysis. Additionally, comprehensive patient background data, including oral care practices and relevant comorbidities such as obesity, physical inactivity, and diet, were not fully documented.

To strengthen future periodontal research, studies should incorporate quantitative bacterial cell analysis alongside detailed assessments of sociodemographic factors and individual oral hygiene practices.

## Conclusions

Geriatric individuals are particularly vulnerable to oral bacterial infections, including *P. gingivalis*, due to the high prevalence of periodontitis and the often asymptomatic nature of its early stages. Regular periodontal screening, even in the absence of symptoms, is essential to prevent severe periodontal destruction and its systemic implications, such as cognitive decline. As Malaysia's elderly population continues to grow, the combination of periodontal disease, chronic conditions, and cardiovascular risk factors is expected to contribute to a rising prevalence of dementia. Therefore, integrating comprehensive non-communicable disease screening, including periodontitis and mild cognitive impairment (MCI) screening, into routine geriatric care is crucial. Our findings support the need for regular MCI and oral microbiome assessments, particularly in elderly individuals with diabetes, and suggest incorporating cognitive screening into existing healthcare diabetic checklist protocol.

## Appendices

**Table 6:** Primers and PCR conditions employed in this study, as used in other previous studies

Primers	PCR condition	Sizes (bp)
PgB1	Initial denaturation was at 50 ^o^C for 2 minutes, followed by 45 cycles for 15s. The annealing temperature was at 55 ^o^C for 30 seconds. The elongation temperature was at 72 ^o^C for 1 minute.	612 - 628
PgP02	The initial denaturation was at 50 ^o^C for 2 minutes, followed by 30 cycles for 30 seconds. The annealing temperature was at 58 ^o^C for 30 seconds. The elongation temperature was at 72 ^o^C for 1 minute, followed by final extension for 10 minutes.	404
PG8Y	The initial denaturation was at 95 ^o^C for 2 minutes, followed by 45 cycles for 5 seconds. The annealing temperature was at 55 ^o^C for 30 seconds. The elongation temperature was at 72 ^o^C for 1 minute.	197
